# Revitalizing neurosurgical frontiers: The EANS frontiers in neurosurgery committee's strategic framework

**DOI:** 10.1016/j.bas.2024.102794

**Published:** 2024-03-26

**Authors:** Aaron Lawson McLean, Ignazio G. Vetrano, Anna C. Lawson McLean, Alfredo Conti, Patrick Mertens, Michael Müther, Jakob Nemir, Simone Peschillo, Antonio Santacroce, Can Sarica, Constantin Tuleasca, Cesare Zoia, Jean Régis, EANS Frontiers in Neurosurgery Committee

**Affiliations:** a Department of Neurosurgery, Jena University Hospital – Friedrich Schiller University Jena, Jena, Germany; b Comprehensive Cancer Center Central Germany (CCCG), Jena University Hospital, Jena, Germany; c Department of Neurosurgery, Fondazione IRCCS Istituto Neurologico Carlo Besta, Milan, Italy; d UOC Neurochirurgia, IRCCS Istituto Delle Scienze Neurologiche di Bologna, Bologna, Italy; e Alma Mater Studiorum Università di Bologna, Bologna, Italy; f Department of Neurosurgery, University Hospital of Neurology and Neurosurgery, Hospices Civils de Lyon, University Lyon 1, Lyon, France; g Department of Neurosurgery, University Hospital Münster, Münster, Germany; h Department of Neurosurgery, University Hospital Center Zagreb, School of Medicine, Zagreb, Croatia; i Endovascular Neurosurgery, Unicamillus-Saint Camillus International University of Health Sciences, Rome, Italy; j Department of Neurosurgery, St. Barbara-Klinik Hamm-Heessen, Hamm, Germany; k Department of Medicine, Faculty of Health, Witten/Herdecke University, Witten, Germany; l European Radiosurgery Center Munich, Munich, Germany; m Division of Neurosurgery, Department of Surgery, University of Toronto, Toronto, Ontario, Canada; n Krembil Research Institute, University Health Network, Toronto, Ontatio, Canada; o Lausanne University Hospital (CHUV), Department of Clinical Neurosciences, Neurosurgery Service and Gamma Knife Center, Lausanne, Switzerland; p University of Lausanne (UNIL), Faculty of Biology and Medicine (FBM), Lausanne, Switzerland; q UOC Neurochirurgia, Ospedale Moriggia Pelascini, Gravedona e Uniti, Italy; r Aix Marseille University, Department of Functional Neurosurgery, CHU Timone, Marseille, France

**Keywords:** Early-career support, Multidisciplinary care, Neurosurgeons, Neurosurgery, Residency, Specialization, Training, Workforce

## Abstract

**Introduction:**

The field of neurosurgery faces challenges with the increasing involvement of other medical specialties in areas traditionally led by neurosurgeons. This paper examines the implications of this development for neurosurgical practice and patient care, with a focus on specialized areas like pain management, peripheral nerve surgery, and stereotactic radiosurgery.

**Research question:**

To assess the implications of the expanded scope of other specialties for neurosurgical practice and to consider the response of the EANS Frontiers in Neurosurgery Committee to these challenges.

**Materials and methods:**

Analysis of recent trends in neurosurgery, including the shift in various procedures to other specialties, demographic challenges, and the emergence of minimally invasive techniques. This analysis draws on relevant literature and the initiatives of the Frontiers in Neurosurgery Committee.

**Results:**

We explore a possible decrease in neurosurgical involvement in certain areas, which may have implications for patient care and access to specialized neurosurgical interventions. The Frontiers in Neurosurgery Committee's role in addressing these concerns is highlighted, particularly in terms of training, education, research, and networking for neurosurgeons, especially those early in their careers.

**Discussion and conclusion:**

The potential decrease in neurosurgical involvement in certain specialties warrants attention. This paper emphasizes the importance of carefully considered responses by neurosurgical societies, such as the EANS, to ensure neurosurgeons continue to play a vital role in managing neurological diseases. Emphasis on ongoing education, integration of minimally invasive techniques, and multidisciplinary collaboration is essential for maintaining the field's competence and quality in patient care.

## Highlights

• Examines the trend of specialties like orthopedic surgery and neuroradiology venturing into traditionally neurosurgical areas.
• Discusses the implications of this ‘scope creep’ for neurosurgical training, expertise maintenance, and patient care quality
• Highlights the demographic challenges in neurosurgery, such as the limited number of practitioners.
• Emphasizes the importance of adopting minimally invasive techniques and technologies to maintain neurosurgery's edge
• Details the EANS Frontiers in Neurosurgery Committee's initiatives for reinforcing neurosurgeons' roles

## 1 Introduction

Neurosurgery is a field that has traditionally played a central role in the diagnosis and management of a wide range of disorders of the central and peripheral nervous system. However, in recent years, there has been a trend towards other specialties, such as neuroradiology and interventional radiology, otolaryngology, orthopedic and plastic surgery, and radiotherapy breaking into areas in which neurosurgeons have historically been leaders, such as such as pain and spasticity management, peripheral nerve surgery, stereotactic radiosurgery, high-intensity focused ultrasound (HIFU) ablation, spinal surgery, orbital surgery, and endovascular and carotid surgery (Jeon and Kwon, 2008; Maniker and Passannante, 2003; Quadri et al., 2018; Rasulić, 2018; Yang et al., 2019). This phenomenon, which could be described as “scope creep” by other specialties, has implications not just for the profession but significantly for patient care, potentially leading to the underutilization of neurosurgical expertise in treating conditions in the aforementioned areas. It also raises broader concerns about the possible decline in the involvement of neurosurgery in these fields, and the potential for neurosurgeons to receive inadequate training and support to maintain excellence in these areas.

This trend can be attributed to several factors, such as the development of new technologies and techniques that makes it possible for other specialists to take on tasks historically undertaken by neurosurgeons, the desire for providers from these specialties to increase revenue streams by offering a broader range of services, a shortage of trained neurosurgeons, and the growing complexity of neurosurgical procedures, necessitating extensive training and resources.

### 1.1 Scope of the problem

The diminishing involvement of neurosurgeons in certain specialties is a concern with profound implications for both the profession and patient outcomes. A notable decline in training and support in these areas may lead to suboptimal patient care. Furthermore, the encroachment of other specialties into these domains risks a significant loss of neurosurgical expertise and may adversely affect the quality and specificity of care. This issue is particularly acute in less traditional or perceived ‘non-authentic’ neurosurgical subspecialties, such as HIFU and radiosurgery (Samuel et al., 2018). The minimally invasive nature of these procedures may dissuade neurosurgeons from engaging in these fields, contributing to a narrowing of the discipline's scope (Ding et al., 2020; Yang et al., 2019).

This decrease in neurosurgical involvement initiates a detrimental cycle: diminished leadership in the field, erosion of specialized competence, loss of expert knowledge, and ultimately, a decline in the standard of patient care. Patients face the most significant repercussions, including restricted access to bespoke neurosurgical interventions and a deficiency in organ-specific expertise, which are crucial for optimal outcomes. Additionally, the dwindling presence of neurosurgeons in these subspecialties curtails the global leadership of the field, impeding the dissemination of specialized neurosurgical skills and knowledge essential for addressing distinct clinical challenges.

The potential complete takeover of these areas by other medical specialties not only raises concerns about the dilution of neurosurgical expertise and the field's integrity but also emphasizes the urgent need for enhanced training and support to uphold the standards of excellence in these evolving domains. This urgency sets the stage for a deeper examination of the landscape, where diminishing involvement, notably in fields such as peripheral nerve surgery, signals a shift towards other specialties augmenting their footprint in traditional neurosurgical territories. Concurrently, the spectrum of neurosurgery is not solely defined by contraction; it also encompasses potential areas of growth, such as the adoption of emerging technologies like HIFU. This juxtaposition of erosion, the gradual retreat from established domains, against expansion, the strategic move towards novel or historically peripheral areas fueled by technological innovation and cross-disciplinary endeavors, encapsulates the dynamic challenges and opportunities facing the neurosurgical field today.

A critical consequence of reduced neurosurgeon involvement in certain specialty areas is the potential detrimental effect on patient care. In multidisciplinary team settings focusing on these subspecialties, the input of experienced neurosurgeons is indispensable (Abussuud et al., 2020; Peck et al., 2020; Rasulić, 2017; Robinson et al., 2022; Staudt, 2022; Zoia et al., 2023). Neurosurgical solutions or insights are often paramount for optimal treatment, and the absence of neurosurgical participation may lead to missed opportunities for ideal patient care, potentially resulting in suboptimal outcomes and patient dissatisfaction. Moreover, the scarcity of neurosurgeons trained in these areas could impede patient access to specialized care, a problem exacerbated in remote or underserved regions where such expertise is scarce yet critical.

In conclusion, the reduced engagement of neurosurgeons in certain specialty areas has wide-ranging repercussions, encompassing inadequate training and support, erosion of neurosurgical expertise, potential negative impacts on patient care, and limitations on access to specialized care. Addressing these challenges necessitates a concerted effort from the EANS and other stakeholders to ensure that neurosurgeons retain an integral role in the management of neurological diseases.

### 1.2 Demographic challenges in neurosurgery: size and subspecialty coverage

Neurosurgery, as a specialty, faces unique demographic challenges rooted in its relatively small size compared to broader fields like orthopedics and anesthesiology (Roche et al., 2019; Reulen et al., 2009; Singh et al., 2022a, Singh et al., 2022b; Ukachukwu et al., 2022). This disparity manifests in a constrained capacity to cultivate and sustain expertise across an expanding range of subspecialties. With fewer neurosurgeons available, the specialty grapples with the dual challenge of ensuring comprehensive coverage in both general and emergency neurosurgical care while striving to develop and maintain proficiency in niche or rare procedures that are increasingly pivotal (Warf, 2013). The limited number of practitioners thus becomes a bottleneck, restricting the potential to fully invest in and foster subspecialty areas. This scenario places additional pressure on neurosurgeons, who must balance the demands of general practice with the pursuit of specialized expertise (Ringel et al., 2023). The need for a strategic approach to training and resource allocation is therefore paramount, ensuring that neurosurgery can adapt and thrive within these constraints, and continue to provide high-quality, specialized care.

Confronting these demographic and specialization challenges, the field of neurosurgery is increasingly recognizing the value of new minimally invasive techniques. Their emergence represents a critical opportunity for the discipline to expand its repertoire and enhance patient care within the confines of its workforce constraints. Neurosurgeons must, therefore, be proactive in integrating these innovations into their practice. This integration extends beyond mere adoption; it necessitates a commitment to developing and participating in comprehensive training programs. Such initiatives are essential not only for mastering current methodologies but also for leading the way in the adoption and refinement of emerging technologies. Embracing these advancements, therefore, becomes a dual imperative: advancing the field's clinical capabilities and ensuring its sustainability in the face of demographic and subspecialty challenges.

### 1.3 Embracing innovation: Neurosurgery's response to minimally invasive advancements

In the evolving landscape of modern medical care, minimally invasive approaches, characterized by reduced recovery times, lower risk of complications, and less postoperative discomfort, are becoming more appealing to patients (Lewandrowski et al., 2020; Narain et al., 2018; White et al., 2022). This trend necessitates a proactive response from neurosurgeons, who must not only acknowledge but actively adapt to these changing preferences.

Each emergence of a new minimally invasive technique in the clinical neurosciences represents a critical juncture. Neurosurgeons should feel compelled to quickly engage with these advancements, entailing the creation of pathways for specialized training and the groundwork necessary for their successful integration into neurosurgical practice. This swift and strategic response is vital not only in meeting patient demand but also in maintaining the neurosurgical profession's leadership and wide competence.

A pertinent example is the current management of HIFU programs in many centers, where neurologists often oversee and operate these devices (Baek et al., 2022; Duc and Keserci, 2019). This scenario, where neurologists are primarily responsible for procedures involving intracranial lesioning, traditionally a domain of neurosurgery, reflects a significant oversight in the neurosurgical field. It underscores the need for neurosurgeons to take a more assertive role in adopting and mastering new technologies. The failure to do so not only diminishes the neurosurgical profession's role in cutting-edge treatments but also potentially compromises the comprehensive care that patients should receive.

Thus, it is essential for neurosurgeons to remain agile and forward-thinking, embracing new technologies and techniques as they arise. This approach ensures that the field of neurosurgery continues to lead in the management of neurological conditions, aligns with patient preferences for minimally invasive options, and upholds the highest standards of patient care.

### 1.4 Technological advancements in neurosurgery: challenges and opportunities for expanding frontiers

The advent of technological advancements in healthcare presents a dual-edged sword for the field of neurosurgery. On one hand, the proliferation of minimally invasive techniques and robotics, alongside advances in imaging and diagnostics, challenges traditional neurosurgical practices by potentially shifting certain procedures to other specialties (Mithany et al., 2023). This phenomenon underscores the necessity for neurosurgeons to continually update their skills and embrace new technologies to remain at the forefront of patient care. On the other hand, these same advancements offer unprecedented opportunities to expand the frontiers of neurosurgery into both familiar and uncharted territories. For example, the integration of artificial intelligence and machine learning can revolutionize the way neurosurgeons approach diagnosis, treatment planning, and risk assessment, leading to more personalized and effective care (Kazemzadeh et al., 2023). Additionally, the development of innovative technologies, such as brain-computer interfaces and advanced neuroprosthetics, opens new avenues for treating neurological disorders that were previously deemed intractable, thus broadening the scope of neurosurgical intervention (Singh et al., 2022a, Singh et al., 2022b; Young et al., 2021). Embracing these opportunities requires a strategic approach, including the adoption of interdisciplinary collaboration and the pursuit of targeted research initiatives to explore the full potential of technological innovations in neurosurgery. By actively engaging with these advancements, the neurosurgical community can ensure its vital role in advancing healthcare and improving patient outcomes in an era of rapid technological change.

### 1.5 Shared and unique challenges across neurosurgical frontiers

An examination of the diverse frontiers in neurosurgery reveals both shared and unique challenges across these subspecialties, as detailed in Table 1. A common thread among many of these areas is the competitive pressure from other medical specialties, which have either historically overlapped with or have recently encroached upon domains traditionally managed by neurosurgeons. For instance, advancements in less invasive techniques in interventional radiology present a shared challenge for areas like carotid and endovascular surgery (Harbaugh and Agarwal, 2006; Peschillo and Delfini, 2012). Similarly, the control and utilization of emerging technologies, such as HIFU, predominantly by departments outside of neurosurgery, such as neurology, radiooncology and radiology, represent a universal challenge across these subspecialties.

**Table 1:** Challenges and competing specialties in the at-risk neurosurgical subspecialty areas identified by the EANS Frontiers in Neurosurgery Committee.

Subspecialty area	Challenges	Competing specialties	Reasons for competition and additional factors
Carotid and endovascular neurosurgery	Advanced techniques dominated by other specialties; Neurosurgeons facing reduced case exposure	Radiology, Cardiology, General vascular surgery	Technological advancements favoring less invasive procedures; Shift in procedural preference towards interventional radiology
High-intensity focused ultrasound (HIFU) ablation	Emerging technology with nascent neurosurgical engagement	Neurology, Radiology	Ownership and control of HIFU devices predominantly by neurology departments; Neurology's earlier adoption and utilization of the technology
Orbital tumor surgery	Niche field with limited neurosurgical emphasis	Ophthalmology, Plastic surgery, Otolaryngology (ENT); Maxillofacial surgery	Confined anatomical area touches various clinical specialties; Rare diseases dispersed among many specialties; Ophthalmology's established domain; Plastic surgery's expertise in reconstructive aspects; ENT and maxillofacial surgery's involvement in adjacent anatomical areas
Pain surgery	Neurosurgical role overshadowed by other approaches; Diverse treatment options	Anesthesiology, Pain management, Radiology	Anesthesiology's dominance of referral pathways; Growth of non-surgical pain management methods
Peripheral nerve surgery	Neurosurgical involvement overshadowed by other surgical fields	Orthopedic surgery, Plastic surgery	Orthopedic surgery's focus on musculoskeletal nerve issues; Plastic surgery's advanced microsurgical techniques
Stereotactic radiosurgery	Technological advancements; Legal and regulatory obstacles	Radiation oncology	Radiation oncology's primary role in radiotherapy
Spasticity surgery	Underappreciation of neurosurgical techniques; Insurance and healthcare system coverage issues	Orthopedic surgery	Lack of awareness about neurosurgical options in spasticity management; Insurance and healthcare systems often do not cover neurosurgical interventions
Spine surgery	Diverse surgical techniques; Competition over surgical vs. conservative management	Orthopedic surgery, Pain medicine, Radiology	Orthopedic surgery's focus on spinal alignment and musculoskeletal issues; Pain medicine's role in conservative management and alternative treatment modalities

However, each frontier also faces its unique set of challenges. In orbital surgery, the competition extends beyond traditional boundaries to include otolaryngology, reflecting the intricate anatomical and functional interplay in this area. Pain surgery faces specific challenges related to the dominance of referral pathways by anesthesiology, impacting patient access to neurosurgical interventions. For spasticity surgery, the primary challenge lies not in competition with other specialties, but in the underappreciation of neurosurgical techniques and the intricacies of coverage by insurers and healthcare systems, especially for implanted devices like pumps. While orthopedic surgery is often considered an alternative, it is, in fact, complementary to neurosurgical approaches. Spine surgery, while facing competition from orthopedic surgery, also grapples with the dichotomy between surgical and conservative management approaches, highlighting the need for a multidisciplinary perspective.

These shared and unique challenges underscore the necessity for tailored strategies in each subspecialty, as well as a unified approach to advocate for the broader neurosurgical field.

Reflecting on past experiences, the interdisciplinary collaborations between neurosurgery and fields such as radiology, oncology, and neurology have been a bedrock for significant advancements and innovations. Lessons learned from these collaborations highlight the value of pooling expertise to tackle complex neurosurgical challenges more effectively. For instance, in spine surgery, the transition from open to minimally invasive techniques has necessitated a shift in training paradigms, reflecting the importance of adaptability in surgical practices. For example, the adoption of robotic-assisted spine surgery presents a learning curve but can ultimately enhance precision and reduce patient recovery times (Vo et al., 2020). In vascular neurosurgery, the shift from surgical aneurysm clipping to endovascular coiling techniques has required neurosurgeons and neuroendovascular surgeons to gain proficiency in catheter-based procedures, underscoring the necessity for ongoing education in emerging technologies (Day et al., 2017). Oncology has seen a similar evolution, with the introduction of intraoperative MRI and fluorescence-guided surgery allowing for more accurate tumor resections (Bin-Alamer et al., 2023). These developments across spine, vascular, and oncological neurosurgery exemplify the need for a multidisciplinary approach, integrating knowledge from radiology, oncology, and neurology, to provide comprehensive patient care and navigate the complexities of modern neurosurgical interventions.

### 1.6 Role of the EANS frontiers in Neurosurgery Committee

The EANS Frontiers in Neurosurgery Committee originated in 2020 as a Task Force aimed at addressing the decline in neurosurgeons’ engagement in specific specialties or “frontiers” (Table 1). Its elevation to Committee status in 2022 reflects the increasing importance of its mission and the expanding scope of its activities. Its primary goal is to bolster training, education, research, and networking among early-career neurosurgeons in Europe, focusing on these areas.

The committee is pursuing an array of proactive strategies and creative solutions to address the challenges faced by the field (Table 2). These include pinpointing unique opportunities for neurosurgeons, offering specialized training and education to enhance their expertise in these specialties, and promoting collaborations with relevant EANS sections and subspecialty societies like the European Society for Stereotactic and Functional Neurosurgery (ESSFN). Efforts to raise awareness about the crucial role of neurosurgeons and advocate for greater recognition of their expertise are also underway.

**Table 2:** Overview of EANS Frontiers in Neurosurgery Committee initiatives. This table provides an overview of the various initiatives under development, detailing their nature, description, and the expected impact on the field of neurosurgery and patient care.

Initiative type	Description	Expected impact
Training programs	Specialized programs focusing on at-risk neurosurgical frontiers	Enhance skill sets and knowledge in emerging and declining neurosurgical areas
Research support	Grants and platforms for innovative neurosurgical research	Foster advancements in neurosurgical techniques and patient care
Networking opportunities	Creation of forums and collaboration platforms for neurosurgeons	Facilitate knowledge exchange and mentorship; Strengthen the neurosurgical community
Advocacy efforts	Engagement with policy-makers and stakeholders	Influence policies to support neurosurgery's role in specialized areas
Global outreach	Partnerships with international neurosurgical societies	Expand the committee's impact globally and ensure inclusive access to resources
Monitoring and evaluation	Implementation of a framework to assess the effectiveness of initiatives	Allow for continuous improvement and alignment with neurosurgical needs

A significant initiative by the committee involves mapping out fellowship and observership opportunities for early-career neurosurgeons in these frontiers, to be hosted on the EANS website. This resource, which will be hosted on the EANS website, will serve as a centralized location for neurosurgeons to find training and educational opportunities in their subspecialty areas of interest. By providing access to these opportunities, the Frontiers in Neurosurgery Committee is helping to ensure that the next generation of neurosurgeons receives the requisite training and support to maintain the crucial role of neurosurgeons in managing a broad range of neurological conditions. Through targeted training, education, and collaborations with other subspecialty societies, the committee is striving to ensure that neurosurgeons acquire and maintain their expertise across key frontiers and that patients receive optimal care.

To constructively address the challenges identified within the neurosurgical landscape, it becomes imperative to delineate a series of actionable strategies that foster resilience and innovation within the field. This necessitates a concerted focus on enhancing neurosurgical training, promoting multidisciplinary collaboration, and optimizing the allocation of resources across varied healthcare systems. For instance, the development of specialized fellowship programs can mitigate subspecialty knowledge gaps, thereby enriching the competency base of the neurosurgical workforce. Furthermore, the establishment of cross-border educational partnerships stands to amplify the dissemination of best practices and cutting-edge techniques, facilitating a more cohesive and integrated European neurosurgical community. Additionally, advocating for policy reforms that encourage sustainable funding models will be crucial in supporting neurosurgical research and the adoption of advanced technologies. Through these multifaceted strategies, the EANS Frontiers in Neurosurgery Committee is committed to reinvigorating the role of neurosurgeons in the identified specialty areas. However, the aim must not only be to address current challenges and reinvigorate the role of neurosurgeons in the identified specialty areas but also endeavor to establish a solid foundation for the advancement of neurosurgery. This initiative is critical for enhancing the field's adaptability and maintaining high standards of patient care in a continuously changing healthcare environment.

### 1.7 Beyond the operating room: neurosurgery in the context of health administration and economics

In addressing the broader contextual framework within which neurosurgery operates, it is imperative to consider the varied landscape of health systems across Europe (Ciulla et al., 2023). The efficiency, structure, and outcomes of neurosurgical interventions are deeply influenced by the diversity of health care systems, which range from publicly funded models to hybrid systems incorporating elements of private funding (Kim et al., 2017; Pham et al., 2022; Venkatesh et al., 2022). This diversity impacts not only access to neurosurgical care but also the availability of resources for training and development within the field (Robertson et al., 2020; Sarpong et al., 2022). European countries exhibit significant variation in their investment in health care, with a correlation to patient outcomes and the capacity for innovation within neurosurgery (Lartigue et al., 2021; Tambor et al., 2021). The strategic allocation of resources towards neurosurgical departments is crucial for fostering advancements in minimally invasive techniques and ensuring that neurosurgeons are at the vanguard of adopting and refining these methods. Moreover, the intricacies of health economics play a pivotal role in shaping the landscape of neurosurgical care, influencing decisions regarding the adoption of new technologies, the allocation of funding for neurosurgical research, and the economic sustainability of specialized neurosurgical interventions (Ryu et al., 2018).

Furthermore, the neurosurgical workforce across Europe faces unique challenges related to training and specialization, underscored by the increasing subspecialization within the field (Ringel et al., 2023). The demographic realities of an aging population and the concurrent rise in neurodegenerative and vascular diseases necessitate a robust response in training the next generation of neurosurgeons (Beard and Bloom, 2015; Edlmann and Whitfield, 2020; Whitehouse et al., 2016). There is a pressing need for educational frameworks that not only impart technical expertise but also foster adaptability and innovation in practice. This entails a comprehensive understanding of health systems management, health economics, and policy, equipping neurosurgeons to navigate the complexities of health care delivery effectively (Castlen et al., 2017). Enhanced collaboration between academic institutions, professional societies, and health care policy makers is essential to address these challenges. By integrating these broader health administration perspectives into the neurosurgical discourse, the EANS Frontiers in Neurosurgery Committee can advocate for policies that support the development of a dynamic and responsive neurosurgical workforce, capable of advancing the field and improving patient outcomes in an ever-evolving health care environment.

The variation in regional practices concerning the involvement in certain neurosurgical frontiers, such as carotid endarterectomy, underscores the diversity of the neurosurgical discipline across different health care contexts (Hannan et al., 2001; LaMuraglia et al., 2004; Uno et al., 2020). Specifically, in regions such as North America and parts of Europe, neurosurgeons often take a leading role in carotid endarterectomy, driven by historical precedents and the integration of vascular surgery into neurosurgical training programs. Conversely, in other regions, such as certain countries in Asia and Eastern Europe, vascular general surgeons or interventional radiologists may predominantly perform these procedures, influenced by local training structures, resource availability, and institutional policies. In France, the establishment of a specialized diploma for stereotactic radiosurgery, endorsed by the French Nuclear Safety Authority (Autorité de Sûreté Nucléaire) and aligned with European Union of Medical Specialists (EUMS) recommendations, exemplifies a structured approach to ensuring the proficiency of neurosurgeons and radiation oncologists in specialized practices in the field of radiosurgery for neurosurgical indications, thereby influencing and reinforcing the scope of their involvement in these procedures within the French healthcare context (Borius et al., 2022). This variation not only reflects the multifaceted nature of neurosurgery as a discipline but also highlights the importance of cross-disciplinary collaboration and the potential for neurosurgeons to either reclaim or further solidify their role in these areas through enhanced training and adaptation to emerging technologies.

This variation not only reflects the multifaceted nature of neurosurgery as a discipline but also highlights the importance of cross-disciplinary collaboration and the potential for neurosurgeons to either reclaim or further solidify their role in these areas through enhanced training and adaptation to emerging technologies.

### 1.8 Strengthening neurosurgery through interdisciplinary collaboration and political advocacy

The political interface and collaboration between specialty organizations will play a crucial role in shaping the future of neurosurgery, by mapping out the terrain for mutual benefits and advancing the field through shared knowledge and resources. Notably, partnerships such as the one between the EANS and the European Board of Neuroradiology (EBNR) have been instrumental in expanding the European Diploma of Interventional Neuroradiology (EDiINR) to include neurosurgeons (Meling and Thurnher, 2024). This expansion is a testament to the foresight and commitment of these organizations to foster interdisciplinary learning and cooperation. By encouraging neurosurgeons to engage in neuroradiology and neuroendovascular training, this collaborative effort has not only broadened the skill set of neurosurgeons but also enhanced the quality of care for patients with neurovascular disorders. Such strategic alliances underscore the importance of a united front in the medical community, advocating for educational reforms, sharing best practices, and working towards common goals. These efforts exemplify how structured collaboration and political advocacy can lead to meaningful advancements in neurosurgery, ensuring the field remains at the cutting edge of medical science and patient care.

## 2 Conclusion

In summary, the formation of the EANS Frontiers in Neurosurgery Committee represents a strategic response to the observed decline in neurosurgeons' engagement in specific specialty areas. The committee's core mission is to identify, cultivate, and create avenues for training, research, and networking tailored to early-career neurosurgeons. This initiative is geared towards reinforcing the role of neurosurgeons in each of the identified frontiers, thereby supporting the broader neurosurgical field in its continuous progression and expansion.

Additionally, concerted efforts are required at both national and international levels within the existing professional societies. These efforts are crucial for articulating and safeguarding the realm of neurosurgical expertise and involvement.

The committee has pinpointed key areas of specialty focus, including neurosurgery for pain and spasticity, radiosurgery, peripheral nerve surgery, spinal surgery, carotid and endovascular neurosurgery, orbital tumor surgery, and HIFU ablation. These areas represent a diverse array of neurological disorders, underscoring the necessity for neurosurgeons to maintain a central role in their diagnosis, evaluation, and treatment.

Through the strategic initiatives and innovative solutions formulated by the committee, such as targeted training programs, the promotion of research, fostering networking opportunities, and partnerships with other subspecialty societies, we aim to ensure that neurosurgeons continue to play a pivotal role in the comprehensive management of a wide spectrum of neurological disorders. This commitment not only seeks to prevent the relinquishment of these areas to other medical specialties but also aspires to fortify the field of neurosurgery, securing its sustained success and growth in the future.

## Authorship statement

ALM led the drafting and development of the manuscript, with contributions from IGV, ACLM and JR. The final version reflects the intellectual input of all named authors, who have approved it for publication. All authors meet ICMJE authorship criteria.

## Funding statement

No external funding was secured for this work.

## Patient consent for publication

Not applicable.

## Ethics approval

Not applicable.

## Declaration of competing interest

The authors declare the following financial interests/personal relationships which may be considered as potential competing interests:

All authors of this article are members of the EANS Frontiers in Neurosurgery Committee. The authors declare no further potential conflicts of interest with respect to the research, authorship, and/or publication of this article.
